# 3-{1-[4-(2-Methyl­prop­yl)phen­yl]eth­yl}-4-phenyl-1*H*-1,2,4-triazole-5(4*H*)-thione

**DOI:** 10.1107/S1600536811025773

**Published:** 2011-07-09

**Authors:** Hoong-Kun Fun, Chin Sing Yeap, K. Manjunath, D. Jagadeesh Prasad, Boja Poojary

**Affiliations:** a X-ray Crystallography Unit, School of Physics, Universiti Sains Malaysia, 11800 USM, Penang, Malaysia; b Department of Chemistry, Mangalore University, Karnataka, India

## Abstract

In the title compound, C_20_H_23_N_3_S, the central 1,2,4-triazole ring makes dihedral angles of 69.76 (9) and 81.69 (8)°, respectively, with the phenyl and benzene rings. In the crystal, mol­ecules are linked into a centrosymmetric dimer by a pair of inter­molecular N—H⋯S hydrogen bonds, generating an *R*_2_^2^(8) ring motif.

## Related literature

For general background to and applications of 1,2,4-triazole derivatives, see: Holla *et al.* (1998 ▶, 2003 ▶); Maxwell *et al.* (1994 ▶); Turan-Zitouni *et al.* (1999 ▶); Demirbas & Demirbas (2002 ▶); Kritsanida *et al.* (2002 ▶); Burch & Smith (1966 ▶); Kalyoncuoglu *et al.* (1992 ▶); Mir *et al.* (1970 ▶). For hydrogen-bond motifs, see: Bernstein *et al.* (1995 ▶).
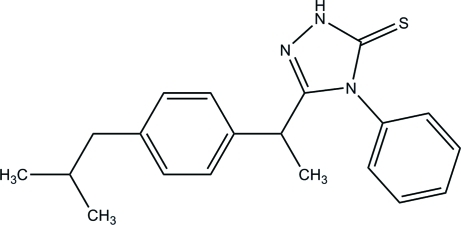


## Experimental

### 

#### Crystal data

C_20_H_23_N_3_S
*M* *_r_* = 337.47
Triclinic, 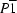

*a* = 6.3249 (2) Å
*b* = 12.4958 (5) Å
*c* = 12.9125 (4) Å
α = 77.649 (1)°
β = 78.133 (1)°
γ = 76.551 (1)°
*V* = 956.44 (6) Å^3^
*Z* = 2
Mo *K*α radiation
μ = 0.18 mm^−1^
*T* = 297 K
0.57 × 0.29 × 0.16 mm

#### Data collection

Bruker APEXII DUO CCD area-detector diffractometer
Absorption correction: multi-scan (*SADABS*; Bruker, 2009 ▶) *T*_min_ = 0.907, *T*_max_ = 0.973
30772 measured reflections
8473 independent reflections
5131 reflections with *I* > 2σ(*I*)
*R*_int_ = 0.029

#### Refinement

*R*[*F* ^2^ > 2σ(*F* ^2^)] = 0.055
*wR*(*F* ^2^) = 0.190
*S* = 1.05
8473 reflections
220 parameters
H-atom parameters constrained
Δρ_max_ = 0.28 e Å^−3^
Δρ_min_ = −0.27 e Å^−3^

### 

Data collection: *APEX2* (Bruker, 2009 ▶); cell refinement: *SAINT* (Bruker, 2009 ▶); data reduction: *SAINT*; program(s) used to solve structure: *SHELXTL* (Sheldrick, 2008 ▶); program(s) used to refine structure: *SHELXTL*; molecular graphics: *SHELXTL*; software used to prepare material for publication: *SHELXTL* and *PLATON* (Spek, 2009 ▶).

## Supplementary Material

Crystal structure: contains datablock(s) global, I. DOI: 10.1107/S1600536811025773/is2744sup1.cif

Structure factors: contains datablock(s) I. DOI: 10.1107/S1600536811025773/is2744Isup2.hkl

Supplementary material file. DOI: 10.1107/S1600536811025773/is2744Isup3.cml

Additional supplementary materials:  crystallographic information; 3D view; checkCIF report

## Figures and Tables

**Table 1:** Hydrogen-bond geometry (Å, °)

*D*—H⋯*A*	*D*—H	H⋯*A*	*D*⋯*A*	*D*—H⋯*A*
N2—H1*N*2⋯S1^i^	0.90	2.43	3.2982 (11)	161

Symmetry code: (i) 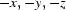.

**Fig. 1.**
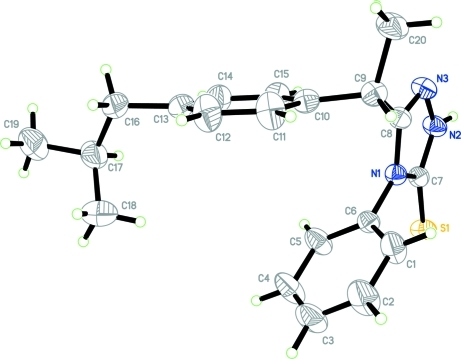
The molecular structure of the title compound, with atom labels and 30% probability ellipsoids for non-H atoms.

**Fig. 2.**
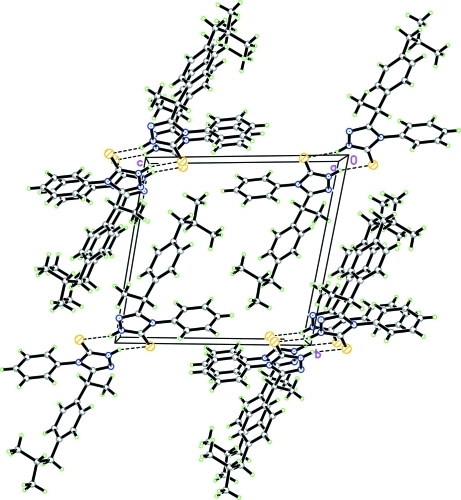
The crystal packing of the title compound, showing the molecules linked into dimers stacked along the a axis. Hydrogen bonds (dashed lines) are shown.

## supplementary crystallographic information

### Comment

1,2,4-Triazole derivatives possess comprehensive bioactivities such as antimicrobial (Holla *et al.*, 1998), anti-inflammatory (Maxwell *et al.*, 1994), analgesic (Turan-Zitouni *et al.*, 1999), antitumor (Demirbas & Demirbas, 2002) and antiviral activities (Kritsanida *et al.*, 2002). Among the 1,2,4-triazoles, the mercapto-thione-substituted 1,2,4-triazole ring systems have been well studied and so far, a variety of biological activities have been reported for a large number of their derivatives, such as antibacterial (Burch & Smith, 1966), antifungal (Kalyoncuoglu *et al.*, 1992), antitubercular (Mir *et al.*, 1970) and anticancer properties (Holla *et al.*, 2003).

The central 1,2,4-triazole ring makes dihedral angles of 69.76 (9) and 81.69 (8)°, respectively, with the phenyl C1–C6 ring and the benzene C10–C15 rings (Fig. 1). In the crystal structure, the molecules are linked into a centrosymmetric dimer by intermolecular N2—H1N2···S1 hydrogen bonds (Table 1 and Fig. 2) generating an *R*_2_^2^(8) ring motif (Bernstein *et al.*, 1995).

### Experimental

A mixture of 2-{2-[4-isobutylphenyl] propanoyl}-*N*-phenylhydrazinecarbothioamide (0.1 mol) and 5% sodium hydroxide (100 ml) was refluxed for 6 h. The reaction mixture was then poured into ice cold water and acidified with dilute hydrochloric acid. The precipitate thus obtained was filtered, dried and re-crystallized from ethanol.

### Refinement

The N-bound hydrogen atom was located in a difference Fourier map and refined using a riding model, with *U*_iso_(H) = 1.2*U*_eq_(N). All C-bound hydrogen atoms were positioned geometrically (C—H = 0.93–0.98 Å) and refined using a riding model, with *U*_iso_(H) = 1.2 or 1.5*U*_eq_(C). A rotating-group model were applied for methyl groups. Five reflections, 0 -3 3, 1 1 5, -1 0 4, -3 -1 6, and 3 5 0, were omitted.

### Crystal data

**Table:**

C_20_H_23_N_3_S	*Z* = 2
*M**_r_* = 337.47	*F*(000) = 360
Triclinic, *P*1	*D*_x_ = 1.172 Mg m^−^^3^
Hall symbol: -P 1	Mo *K*α radiation, λ = 0.71073 Å
*a* = 6.3249 (2) Å	Cell parameters from 7323 reflections
*b* = 12.4958 (5) Å	θ = 3.4–33.2°
*c* = 12.9125 (4) Å	µ = 0.18 mm^−^^1^
α = 77.649 (1)°	*T* = 297 K
β = 78.133 (1)°	Block, yellow
γ = 76.551 (1)°	0.57 × 0.29 × 0.16 mm
*V* = 956.44 (6) Å^3^	

### Data collection

**Table:**

Bruker APEXII DUO CCD area-detector diffractometer	8473 independent reflections
Radiation source: fine-focus sealed tube	5131 reflections with *I* > 2σ(*I*)
graphite	*R*_int_ = 0.029
φ and ω scans	θ_max_ = 35.3°, θ_min_ = 1.7°
Absorption correction: multi-scan (*SADABS*; Bruker, 2009)	*h* = −10→10
*T*_min_ = 0.907, *T*_max_ = 0.973	*k* = −20→20
30772 measured reflections	*l* = −20→20

### Refinement

**Table:**

Refinement on *F*^2^	Primary atom site location: structure-invariant direct methods
Least-squares matrix: full	Secondary atom site location: difference Fourier map
*R*[*F*^2^ > 2σ(*F*^2^)] = 0.055	Hydrogen site location: inferred from neighbouring sites
*wR*(*F*^2^) = 0.190	H-atom parameters constrained
*S* = 1.05	*w* = 1/[σ^2^(*F*_o_^2^) + (0.0928*P*)^2^ + 0.0747*P*] where *P* = (*F*_o_^2^ + 2*F*_c_^2^)/3
8473 reflections	(Δ/σ)_max_ < 0.001
220 parameters	Δρ_max_ = 0.28 e Å^−^^3^
0 restraints	Δρ_min_ = −0.27 e Å^−^^3^

### Special details

**Table:**

Geometry. All e.s.d.'s (except the e.s.d. in the dihedral angle between two l.s. planes) are estimated using the full covariance matrix. The cell e.s.d.'s are taken into account individually in the estimation of e.s.d.'s in distances, angles and torsion angles; correlations between e.s.d.'s in cell parameters are only used when they are defined by crystal symmetry. An approximate (isotropic) treatment of cell e.s.d.'s is used for estimating e.s.d.'s involving l.s. planes.
Refinement. Refinement of *F*^2^ against ALL reflections. The weighted *R*-factor *wR* and goodness of fit *S* are based on *F*^2^, conventional *R*-factors *R* are based on *F*, with *F* set to zero for negative *F*^2^. The threshold expression of *F*^2^ > σ(*F*^2^) is used only for calculating *R*-factors(gt) *etc*. and is not relevant to the choice of reflections for refinement. *R*-factors based on *F*^2^ are statistically about twice as large as those based on *F*, and *R*- factors based on ALL data will be even larger.

### Fractional atomic coordinates and isotropic or equivalent isotropic displacement parameters (Å^2^)

**Table:**

	*x*	*y*	*z*	*U*_iso_*/*U*_eq_	
S1	−0.08075 (6)	−0.01849 (3)	0.18539 (3)	0.05785 (12)	
N1	0.20563 (16)	0.11866 (8)	0.18008 (7)	0.04246 (19)	
N2	0.20973 (18)	0.07739 (9)	0.02809 (8)	0.0521 (2)	
H1N2	0.1912	0.0441	−0.0240	0.062*	
N3	0.35825 (18)	0.14653 (10)	0.00916 (9)	0.0543 (2)	
C1	0.2972 (3)	0.08271 (16)	0.35969 (12)	0.0758 (5)	
H1A	0.4358	0.0431	0.3351	0.091*	
C2	0.2366 (4)	0.0957 (2)	0.46651 (14)	0.0993 (8)	
H2A	0.3370	0.0649	0.5135	0.119*	
C3	0.0349 (3)	0.15220 (18)	0.50391 (13)	0.0847 (6)	
H3A	−0.0044	0.1580	0.5763	0.102*	
C4	−0.1105 (3)	0.2006 (2)	0.43437 (14)	0.0882 (6)	
H4A	−0.2477	0.2416	0.4589	0.106*	
C5	−0.0538 (2)	0.18870 (16)	0.32681 (12)	0.0683 (4)	
H5A	−0.1532	0.2210	0.2795	0.082*	
C6	0.14823 (19)	0.12949 (9)	0.29125 (9)	0.0447 (2)	
C7	0.1115 (2)	0.05877 (9)	0.13045 (9)	0.0444 (2)	
C8	0.35300 (19)	0.17053 (10)	0.10269 (9)	0.0465 (2)	
C9	0.4782 (2)	0.24956 (11)	0.12384 (12)	0.0538 (3)	
H9A	0.5611	0.2109	0.1820	0.065*	
C10	0.3240 (2)	0.35217 (11)	0.15951 (11)	0.0514 (3)	
C11	0.3736 (3)	0.40631 (16)	0.23145 (16)	0.0768 (5)	
H11A	0.4992	0.3759	0.2628	0.092*	
C12	0.2420 (3)	0.50401 (16)	0.25793 (18)	0.0815 (5)	
H12A	0.2810	0.5382	0.3065	0.098*	
C13	0.0531 (2)	0.55268 (11)	0.21409 (13)	0.0621 (3)	
C14	−0.0013 (3)	0.49653 (14)	0.14492 (14)	0.0685 (4)	
H14A	−0.1302	0.5254	0.1160	0.082*	
C15	0.1316 (3)	0.39832 (13)	0.11773 (12)	0.0628 (3)	
H15A	0.0909	0.3629	0.0706	0.075*	
C16	−0.0877 (3)	0.66265 (13)	0.23725 (17)	0.0775 (5)	
H16A	−0.0122	0.6946	0.2777	0.093*	
H16B	−0.1012	0.7132	0.1695	0.093*	
C17	−0.3145 (3)	0.65684 (16)	0.29847 (17)	0.0830 (5)	
H17A	−0.3827	0.6169	0.2609	0.100*	
C18	−0.3071 (7)	0.5916 (2)	0.4111 (2)	0.1563 (16)	
H18A	−0.4547	0.5920	0.4490	0.234*	
H18B	−0.2301	0.6254	0.4481	0.234*	
H18C	−0.2319	0.5159	0.4077	0.234*	
C19	−0.4555 (4)	0.7724 (2)	0.2994 (2)	0.1071 (8)	
H19A	−0.4593	0.8111	0.2268	0.161*	
H19B	−0.3944	0.8132	0.3371	0.161*	
H19C	−0.6025	0.7664	0.3347	0.161*	
C20	0.6445 (3)	0.28229 (17)	0.02293 (16)	0.0785 (5)	
H20A	0.7416	0.2161	0.0025	0.118*	
H20B	0.7290	0.3299	0.0384	0.118*	
H20C	0.5664	0.3212	−0.0348	0.118*	

### Atomic displacement parameters (Å^2^)

**Table:**

	*U*^11^	*U*^22^	*U*^33^	*U*^12^	*U*^13^	*U*^23^
S1	0.0759 (2)	0.0634 (2)	0.04339 (17)	−0.03438 (17)	−0.00472 (14)	−0.01132 (13)
N1	0.0465 (5)	0.0467 (5)	0.0371 (4)	−0.0112 (4)	−0.0079 (3)	−0.0105 (3)
N2	0.0607 (6)	0.0613 (6)	0.0401 (5)	−0.0211 (5)	−0.0017 (4)	−0.0180 (4)
N3	0.0545 (6)	0.0665 (6)	0.0446 (5)	−0.0202 (5)	0.0000 (4)	−0.0140 (5)
C1	0.0783 (10)	0.0904 (11)	0.0531 (8)	0.0225 (8)	−0.0283 (7)	−0.0263 (7)
C2	0.1055 (14)	0.1307 (17)	0.0557 (9)	0.0281 (13)	−0.0401 (10)	−0.0338 (10)
C3	0.0979 (13)	0.1126 (14)	0.0452 (7)	−0.0087 (11)	−0.0106 (8)	−0.0313 (9)
C4	0.0690 (10)	0.1325 (17)	0.0594 (9)	0.0050 (10)	−0.0025 (8)	−0.0440 (10)
C5	0.0541 (7)	0.0994 (11)	0.0485 (7)	0.0027 (7)	−0.0115 (6)	−0.0237 (7)
C6	0.0515 (6)	0.0486 (5)	0.0372 (5)	−0.0106 (4)	−0.0097 (4)	−0.0112 (4)
C7	0.0522 (6)	0.0451 (5)	0.0386 (5)	−0.0112 (4)	−0.0075 (4)	−0.0118 (4)
C8	0.0440 (6)	0.0524 (6)	0.0442 (5)	−0.0114 (4)	−0.0063 (4)	−0.0098 (4)
C9	0.0451 (6)	0.0584 (7)	0.0622 (7)	−0.0169 (5)	−0.0124 (5)	−0.0093 (5)
C10	0.0509 (6)	0.0551 (6)	0.0541 (7)	−0.0213 (5)	−0.0118 (5)	−0.0074 (5)
C11	0.0578 (8)	0.0883 (11)	0.1032 (13)	−0.0129 (7)	−0.0314 (9)	−0.0415 (10)
C12	0.0699 (10)	0.0860 (11)	0.1100 (14)	−0.0250 (8)	−0.0201 (9)	−0.0476 (11)
C13	0.0638 (8)	0.0529 (7)	0.0696 (8)	−0.0249 (6)	0.0022 (6)	−0.0089 (6)
C14	0.0738 (9)	0.0646 (8)	0.0661 (9)	−0.0039 (7)	−0.0249 (8)	−0.0072 (7)
C15	0.0684 (8)	0.0658 (8)	0.0600 (8)	−0.0090 (7)	−0.0266 (7)	−0.0126 (6)
C16	0.0772 (10)	0.0588 (8)	0.0919 (12)	−0.0259 (7)	0.0129 (9)	−0.0153 (8)
C17	0.0817 (11)	0.0830 (11)	0.0885 (13)	−0.0356 (9)	0.0170 (9)	−0.0327 (9)
C18	0.206 (4)	0.110 (2)	0.105 (2)	−0.033 (2)	0.059 (2)	0.0048 (16)
C19	0.0893 (14)	0.1154 (17)	0.1069 (17)	−0.0089 (13)	0.0160 (13)	−0.0416 (14)
C20	0.0566 (8)	0.0913 (11)	0.0899 (12)	−0.0339 (8)	0.0087 (8)	−0.0195 (9)

### Geometric parameters (Å, °)

**Table:**

S1—C7	1.6766 (12)	C11—C12	1.374 (2)
N1—C7	1.3766 (13)	C11—H11A	0.9300
N1—C8	1.3815 (15)	C12—C13	1.382 (2)
N1—C6	1.4341 (14)	C12—H12A	0.9300
N2—C7	1.3361 (15)	C13—C14	1.384 (2)
N2—N3	1.3716 (15)	C13—C16	1.506 (2)
N2—H1N2	0.9023	C14—C15	1.385 (2)
N3—C8	1.2975 (15)	C14—H14A	0.9300
C1—C6	1.3695 (17)	C15—H15A	0.9300
C1—C2	1.388 (2)	C16—C17	1.498 (2)
C1—H1A	0.9300	C16—H16A	0.9700
C2—C3	1.354 (3)	C16—H16B	0.9700
C2—H2A	0.9300	C17—C18	1.511 (4)
C3—C4	1.366 (3)	C17—C19	1.512 (3)
C3—H3A	0.9300	C17—H17A	0.9800
C4—C5	1.392 (2)	C18—H18A	0.9600
C4—H4A	0.9300	C18—H18B	0.9600
C5—C6	1.3620 (19)	C18—H18C	0.9600
C5—H5A	0.9300	C19—H19A	0.9600
C8—C9	1.4982 (17)	C19—H19B	0.9600
C9—C10	1.5145 (19)	C19—H19C	0.9600
C9—C20	1.543 (2)	C20—H20A	0.9600
C9—H9A	0.9800	C20—H20B	0.9600
C10—C11	1.3808 (19)	C20—H20C	0.9600
C10—C15	1.3836 (18)		
			
C7—N1—C8	107.80 (9)	C11—C12—C13	121.61 (15)
C7—N1—C6	125.16 (10)	C11—C12—H12A	119.2
C8—N1—C6	126.94 (10)	C13—C12—H12A	119.2
C7—N2—N3	113.85 (10)	C12—C13—C14	116.87 (14)
C7—N2—H1N2	124.5	C12—C13—C16	122.73 (16)
N3—N2—H1N2	121.5	C14—C13—C16	120.39 (15)
C8—N3—N2	104.11 (10)	C13—C14—C15	121.57 (14)
C6—C1—C2	118.49 (15)	C13—C14—H14A	119.2
C6—C1—H1A	120.8	C15—C14—H14A	119.2
C2—C1—H1A	120.8	C10—C15—C14	121.04 (14)
C3—C2—C1	121.58 (15)	C10—C15—H15A	119.5
C3—C2—H2A	119.2	C14—C15—H15A	119.5
C1—C2—H2A	119.2	C17—C16—C13	115.34 (13)
C2—C3—C4	119.39 (15)	C17—C16—H16A	108.4
C2—C3—H3A	120.3	C13—C16—H16A	108.4
C4—C3—H3A	120.3	C17—C16—H16B	108.4
C3—C4—C5	120.11 (16)	C13—C16—H16B	108.4
C3—C4—H4A	119.9	H16A—C16—H16B	107.5
C5—C4—H4A	119.9	C16—C17—C18	111.5 (2)
C6—C5—C4	119.58 (14)	C16—C17—C19	111.03 (16)
C6—C5—H5A	120.2	C18—C17—C19	111.5 (2)
C4—C5—H5A	120.2	C16—C17—H17A	107.5
C5—C6—C1	120.81 (12)	C18—C17—H17A	107.5
C5—C6—N1	119.09 (10)	C19—C17—H17A	107.5
C1—C6—N1	120.09 (11)	C17—C18—H18A	109.5
N2—C7—N1	103.26 (10)	C17—C18—H18B	109.5
N2—C7—S1	128.48 (9)	H18A—C18—H18B	109.5
N1—C7—S1	128.26 (9)	C17—C18—H18C	109.5
N3—C8—N1	110.98 (10)	H18A—C18—H18C	109.5
N3—C8—C9	124.99 (12)	H18B—C18—H18C	109.5
N1—C8—C9	123.92 (11)	C17—C19—H19A	109.5
C8—C9—C10	111.30 (10)	C17—C19—H19B	109.5
C8—C9—C20	110.08 (12)	H19A—C19—H19B	109.5
C10—C9—C20	110.99 (12)	C17—C19—H19C	109.5
C8—C9—H9A	108.1	H19A—C19—H19C	109.5
C10—C9—H9A	108.1	H19B—C19—H19C	109.5
C20—C9—H9A	108.1	C9—C20—H20A	109.5
C11—C10—C15	117.19 (14)	C9—C20—H20B	109.5
C11—C10—C9	121.54 (12)	H20A—C20—H20B	109.5
C15—C10—C9	121.21 (12)	C9—C20—H20C	109.5
C12—C11—C10	121.64 (14)	H20A—C20—H20C	109.5
C12—C11—H11A	119.2	H20B—C20—H20C	109.5
C10—C11—H11A	119.2		
			
C7—N2—N3—C8	0.55 (15)	C6—N1—C8—C9	−0.56 (18)
C6—C1—C2—C3	0.6 (4)	N3—C8—C9—C10	113.63 (14)
C1—C2—C3—C4	−2.1 (4)	N1—C8—C9—C10	−62.26 (15)
C2—C3—C4—C5	2.1 (4)	N3—C8—C9—C20	−9.86 (18)
C3—C4—C5—C6	−0.7 (3)	N1—C8—C9—C20	174.25 (13)
C4—C5—C6—C1	−0.8 (3)	C8—C9—C10—C11	147.05 (15)
C4—C5—C6—N1	−179.42 (17)	C20—C9—C10—C11	−89.98 (18)
C2—C1—C6—C5	0.8 (3)	C8—C9—C10—C15	−35.72 (18)
C2—C1—C6—N1	179.43 (18)	C20—C9—C10—C15	87.25 (16)
C7—N1—C6—C5	−68.30 (17)	C15—C10—C11—C12	−2.2 (3)
C8—N1—C6—C5	107.57 (15)	C9—C10—C11—C12	175.17 (16)
C7—N1—C6—C1	113.07 (16)	C10—C11—C12—C13	0.2 (3)
C8—N1—C6—C1	−71.06 (18)	C11—C12—C13—C14	2.0 (3)
N3—N2—C7—N1	−0.83 (14)	C11—C12—C13—C16	−176.77 (17)
N3—N2—C7—S1	178.99 (9)	C12—C13—C14—C15	−2.3 (2)
C8—N1—C7—N2	0.77 (12)	C16—C13—C14—C15	176.50 (15)
C6—N1—C7—N2	177.31 (10)	C11—C10—C15—C14	1.8 (2)
C8—N1—C7—S1	−179.06 (9)	C9—C10—C15—C14	−175.50 (14)
C6—N1—C7—S1	−2.52 (17)	C13—C14—C15—C10	0.4 (3)
N2—N3—C8—N1	−0.02 (13)	C12—C13—C16—C17	−114.2 (2)
N2—N3—C8—C9	−176.36 (12)	C14—C13—C16—C17	67.1 (2)
C7—N1—C8—N3	−0.49 (13)	C13—C16—C17—C18	65.7 (3)
C6—N1—C8—N3	−176.95 (11)	C13—C16—C17—C19	−169.34 (19)
C7—N1—C8—C9	175.90 (11)		

### Hydrogen-bond geometry (Å, °)

**Table:**

*D*—H···*A*	*D*—H	H···*A*	*D*···*A*	*D*—H···*A*
N2—H1N2···S1^i^	0.90	2.43	3.2982 (11)	161

Symmetry codes: (i) −*x*, −*y*, −*z*.
